# The Intergenerational Transmission of Mental and Physical Health in Australia: Evidence Using Data From the Household Income and Labor Dynamics of Australia Survey

**DOI:** 10.3389/fpubh.2021.763589

**Published:** 2022-02-23

**Authors:** Esperanza Vera-Toscano, Heather Brown

**Affiliations:** ^1^Melbourne Institute, Applied Economic and Social Research, The University of Melbourne, Melbourne, VIC, Australia; ^2^Population Health Sciences Institute, Newcastle University, and Fuse-Centre for Translational Research in Public Health, Newcastle upon Tyne, United Kingdom

**Keywords:** intergenerational health correlation, mental health, physical health, QALYs, HILDA Survey, Australia

## Abstract

Promoting good health across the life course is high on countries agenda. There is a growing evidence base that health is correlated across generations. We examine the persistence of physical and mental health status across generations and explore how different early life factors and adult outcomes impact on this association. In particular, we focus on childhood disadvantage and childhood health, educational attainment, and social mobility measured by household income compared to one's parents. We use data from 19 waves of the Household, Income and Labor Dynamics in Australia (HILDA) Survey. The analysis is restricted to young adults (aged 25–35 years old in 2019) and their parents. We find an intergenerational correlation in health which ranges from 0.19 for physical health to 0.20 for the QALY and 0.21 for mental health. After we include covariates related to childhood disadvantage, childhood health, educational attainment, and social mobility, the intergenerational correlations are reduced to 0.13 for physical health, 0.18 for mental health, and 0.14 for QALYs. We find that early life disadvantage is the only factor influencing the intergenerational correlation for all health measures. Policy focusing on reducing the negative impact of early life disadvantage is likely to have a larger impact on improving health across the life course and reducing intergenerational health inequalities.

## Introduction

A healthy population is an essential component of economic growth. In fact, good health across the life course and a strong public health system promoting preventive care and the equitable distribution of resources are critical factors to reducing the negative impact of infectious diseases such as recent Covid-19 (1). Thus, investing in health may be one mechanism to help reduce inequalities and improve social mobility (2).

A growing body of literature treats health inequality as an intraindividual process showing that poor health in childhood is associated with reduced educational attainment and subsequently lower wages in adulthood (3–5). Accordingly, poor health in childhood is also associated with an increased risk of poor health in adulthood (6, 7). However, an important aspect of life course research is the importance of linked lives and the intergenerational context of life course processes (8).

Policymakers and academics (more broadly) have increased their interest in social inequalities in health and in understanding how health is linked between generations and how this relates to the provision of health services and population health.

There is an increasing evidence base of research estimating intergenerational health correlations. From a lower middle-income country setting, Kim et al. (9) found for Indonesia that a father reporting being in poor health increased the likelihood that his daughter reported being in poor health by 29%. Pascual and Cantarero (10), using Spanish data from the European Community Household Panel, found a correlation of between 5 and 10% of parents and children both reporting being in good or excellent health. Johnston et al. (11) using British data from the British Cohort Survey, found a correlation in mental health of ~0.16 after controlling for child health status at age 5, parental socio-economic status, and child cognitive development across three generations. The correlation in mental health between mothers and daughters was ~30% stronger than the correlation between mothers and sons. Halliday et al. (12), using USA data from the Panel Study of Income Dynamics, found an intergenerational correlation in self-reported health of 0.20–0.25. The correlation in poor self-reported health was stronger for families where the parents have lower educational qualifications, and 40% of the observed correlation can be explained by early life circumstances. Bencsik et al. (13) follow a similar approach to Halliday et al. (12) but use data from the British Household Panel Survey and its successor survey, the Understanding Society Survey which includes a richer set of covariates. They find an intergenerational correlation in health of 0.21. When separating mental health from physical health, the intergenerational correlation in mental health was 0.22, and the intergenerational correlation in physical health was 0.17. These results suggest that parental mental health is a stronger predictor of offspring health than parental physical health.

Existing research suggests that there is a significant intergenerational relationship in health which is present across multiple generations. Empirical results show that the relationship between health across generations is stronger for those from lower socio-economic backgrounds (3, 14, 15). The intergenerational correlation in mental health is stronger than the intergenerational correlation in physical health (2, 13). Moreover, the variation in intergenerational correlations in health across different countries (2, 9, 10, 12, 13) suggest that macro level factors may explain some of the variations between studies. The health care, welfare system, and health inequalities may partially explain health mobility (strength of the intergenerational correlation) between generations. Countries with universal health coverage such as the UK have slightly higher health mobility compared to the USA.

To create a resilient population, we need to understand the relationship between health across generations and the factors that influence these relationships.

This paper aims to estimate the intergenerational correlation in three health measures and explore how disadvantage may explain this correlation for young adults aged 25–35 and their parents in Australia using the 19 available waves of the Household, Income and Labor Dynamics of Australia (HILDA) Survey. To achieve this aim, we address the following research questions:

**RQ1**: What is the correlation in physical and mental health between adult offspring aged between 25 and 35 in 2019 and their parents?

**RQ2**: What is the contribution of the role of early life disadvantage, poor childhood health and low educational attainment to the intergenerational correlation in health? and

**RQ3:** To what extent socio-economic mobility (divergence in household income between parents and offspring) contributes to our understanding of the influence of the health of the previous generation?

Australia is a country that has similarities to the UK, such as a universal health care system and similarities to the USA, such as a large geographical landmass, heterogeneous majority immigrant population (16). Thus, we add to the evidence base by comparing the intergenerational correlations in health across these three countries. Our results will provide further evidence to understand if contextual factors play a role in explaining intergenerational correlations in health. By exploring the role of early life factors and adult outcomes on the intergenerational correlation, we provide evidence on potential areas for interventions to improve health mobility and reduce health inequalities.

## Theoretical Framework

Cumulative disadvantage theory (17) provides a suitable ground that helps to formulate our specific research questions. In particular, its life course perspective (8) emphasizes how early advantage or disadvantage is critical to how groups of individuals become segregated over time. Some persons are advantaged in their early years, and this advantage may compound over time. Others are disadvantaged because of genetic or environmental factors, and these disadvantages also accumulate (18, 19). Thus, the relative position early in life produces further benefits or losses across the life course, resulting in the over-time accumulation of advantages for one individual or group relative to another (20, 21).

While initially employed to examine the accumulation of financial resources and socio-economic status, cumulative disadvantage theory has also been used to explore adult health outcomes. There has been an increase of research interested in the accumulative effects of early environment and exposures to disadvantage on later life health, as well as research on the mechanisms between disadvantage and health issues such as stress or health behaviors [see e.g., (22–26)]. Yet, studies using this framework to explore the intergenerational transmission of health inequality across generations within families are limited. More specifically, some authors have focused on the role of childhood health as a “mechanism through which socio-economic status is transferred across generations” [(27), p. 339], while others have placed their attention on offspring having experienced disadvantaged socio-economic origins on top of poor childhood health (3, 28–30). In addition, there has also been some recent interest in the role of the health of one generation on the educational attainment of the next. Thus, Boardman et al. (31) find that parents' poor midlife health leads to lower educational attainment of their offspring, which could be due in part to health's effect on labor market attachment and resources available to support offspring's needs. Other explanations for intergenerational continuity include the transmission of health behaviors [e.g., (32, 33)] and the social contexts in which both health and socio-economic status are practiced and reproduced (31, 34, 35).

In this study, we integrate previous conceptual and empirical work to consider health as a form of life course capital that may be transmitted across generations. Health has the potential to negatively affect one's own life outcomes and the next generation's childhood health, educational attainment, and subsequent midlife health and other life outcomes. Hence, we examine potential explanations for the intergenerational persistence of health, including the role of early-life disadvantage, the role of poor childhood health, and low educational attainment. In addition, we examine the extent to which socio-economic mobility across generations within families contributes to our understanding of the influence of the health of the previous generation. Childhood is the critical time for the development of cognitive and non-cognitive skills, as well as for building adulthood expectations. Thus, the difference (or similarity) between a parent's own childhood and offspring socio-economic status is an important reference for understanding a child's household environment.

We assume that parental investments and the shared family environment in childhood will strengthen correlations. However, young adults' investment in their own health may reduce or strengthen the correlation with their parents' health depending upon how closely young adult offspring follow their parent's preferences for investing in health.

To understand how early life factors and young adult characteristics that may influence health outcomes impact on the correlation in health, we estimate stepwise models that include these characteristics. We hypothesize that controlling for early life factors will reduce some of the correlation in the family correlation in health. We further assume that when there is homophily in parent and offspring adult characteristics, including these offspring's adult characteristics will reduce the correlation. If there is a divergence between parent and their offspring, including these characteristics may not reduce or impact the observed correlation. So overall, when looking at the sample, these characteristics may have no impact on the observed correlation depending upon the degree of homophily/divergence. We assume that this relationship will work similarly for mental and physical health.

After including factors related to childhood and offspring's adulthood, the observed correlation will consist of any unobserved factors not included in our model, such as genetics which may impact the correlation in health. By having these factors in our model, our results will be comparable to other studies in the literature, such as Halliday et al. (12); Brown (2), and Bencsik et al. (13), to compare the intergenerational correlations in health across the USA, UK, and Australia.

## Materials and Methods

We use data from the Household, Income and Labor Dynamics in Australia (HILDA) Survey, a household panel study collecting annual information from individuals from the same households since 2001. The HILDA Survey sample was drawn following a complex, probabilistic design and is largely representative of the population age 15 and older in Australia (36). In total, the HILDA Survey has been running annually for 19 years, making it the longest running annual longitudinal social research study in Australia. We construct an intergenerational sample of parents and their adult offspring. It comprises 1,960 offspring aged 7–17 in 2001 and who are between 25 and 35 years old in 2019, who provide information on their health status as adults (aged 18 or over) in at least one survey year, and who are matched to at least one parent who also provides health information at least once when the offspring was a child^1^. The long-running nature of the HILDA and the possibility to link up household members when they are no longer living together and relatively low attrition rates over time provide us with a unique opportunity to explore the intergenerational correlations in mental and physical health.

As our focus is on the persistence of health status across generations, we use the SF-36 Health Survey (37) to construct three health measures. First, we use an index of physical health that is based on 10 questions in the SF-36. These relate to limitations on activities of daily living and work due to problems with physical health^2^. Raw scores were transformed to a 0–100 scale. A person-specific raw score was estimated for any scale on which there were valid responses on greater than or equal to half the items. The average was calculated and applied to missing data for these individuals. Our second measure is an index of mental health that uses five questions from the SF-36. These questions evaluate the extent to which mental health problems emotionally affect daily activities. The mental health index is calculated in the same way as the physical health index and varies between 0 and 100, with higher values corresponding to better mental health. Finally, The SF-36 includes a question on general health status that is widely collected in many surveys such as the Panel Study of Income Dynamics, USA or Understanding Society Survey, UK and is often referred to as “Self-reported Health Status” (SRHS). The question asked respondent “In general, would you say your health is excellent, very good, good, fair, or poor.” The responses are then coded as a categorical variable. SRHS has been validated as a strong predictor of mortality and hospitalization (38, 39). Following both Halliday et al. (12) and Johnson and Schoeni (40), we converted the categorical values into a continuous measure using health utility-based scale developed for the Health and Activity Limitation Index (HALex). This approach is designed to control for the quality of health (i.e., having good vs. poor health) and is called a QALY-quality adjusted life year. The value ranges for each health status category are as follows: excellent is [95,100]; very good is [85, 95]; good is [70, 85]; fair is [30, 70]; and poor is [1, 30]. We assign the midpoint of the interval for each reported health category in each year and then average these values over all available years for each individual following the method employed by Halliday et al. (12). Using the QALY will also allow us to compare our findings to other studies such as Halliday et al. (12) for the USA and Bencsik et al. (13) for the UK to provide further cross-country evidence on intergenerational health correlations.

### Analytical Strategy

We estimate the intergenerational persistence in health status after controlling for a number of childhood characteristics and socio-economic circumstances as a young adult between the ages of 25–35. To do this, we estimate the following linear model:


(1) $$\begin{array}{l} y_{i}^{C}=\alpha +\beta y_{i}^{P}+X_{i}\theta +\epsilon_{i} \end{array}$$

where $y_{i}^{C}$ and $y_{i}^{P}$are measurements of the health status of the offspring between the ages of 25–35 and the parents (when the offspring was <18 years of age) and *X*_*i*_ includes a set of covariates identified in the literature as potential explanations for the intergenerational persistence of health. These include the role of early-life disadvantage, the role of poor childhood health, and low educational attainment, as well as the impact of income across generations. Previous studies have emphasized the value of using averages over time to estimate latent health status (2, 12, 13). This is analogous to the income mobility literature where controlling for income over time can be used as an approximately measure of permanent or lifetime income, otherwise estimates suffer from attenuation bias (41, 42). When $y_{i}^{C}$ and $y_{i}^{P}$are averages, β measures the extent to which there is an association between parents and offspring health status. Using stepwise models that add the different covariates identified (*X*_*i*_) we can address our three research questions: (1) what is the intergenerational correlation in mental and physical health [RQ1] and (2) how much each of the covariates considered contribute to the intergenerational correlation^3^? [RQ2 and RQ3].

### Dependent Variable

#### Offspring Health Status

To estimate the association between parents and offspring health status, we use offspring health status (whether physical, mental or SRHS) as the time-averaged for all available health observations since the offspring turned 18 years old.

### Independent Variables

#### Parents' Health Status

As for offspring health status, parents' health status is the time-averaged of all available health measures before the offspring turns 18. Our preferred estimates combine the health status of both parents (when available) by using an average of the time averages of each parent and using just a single parent's health measure when only one parent's data is available.

#### Early Life-Disadvantage

Indicators of disadvantage in childhood are measured from the information provided by the parents before the offspring were age 18. These covariates include whether the father and the mother were *ever unemployed and had less than a high school education* and if the family lived in an area of high socio-economic deprivation.

#### Childhood Health

A retrospective question was asked to the respondent to rate their health during childhood. A binary variable was created that was equal to one if the respondent reported poor health in childhood and was equal to zero otherwise. Because this variable has more missing cases than other variables in the analysis, we include a variable indicating a missing value on childhood health to retain these respondents in the analysis and examine whether missing cases are associated with the dependent variable (*childhood health missing* = 1).

#### Income Compared to Childhood Income

Following Wightman and Danziger (43), we compare income in childhood and adulthood using the average annual percentile ranking in the distribution of the parents' equivalised real household disposable income (when the offspring was <18 years old) and offspring once they are >18 years old. At each relevant wave, we divide total household income from all sources by the number of household members and calculate the distribution of this value, weighted with the appropriate year's weight, to produce an annual percentile ranking. We then average the annual percentiles over the relevant age ranges (for parents' income, we use the years the offspring was below 18; for the offspring's income we use the years the offspring are aged 25–35). Relatively low income reflects an average percentile ranking of <50 (below the median), and relatively high income is an average income percentile ranking that is ≥50 (above the median). Then we create four mutually exclusive categories:

- *Stable low-income* respondents whose average ranking falls below the median in adulthood and who resided in households with average income below the median as children.
- *Stable high-income* respondents are those who were raised in a household with income above the median and who on average have income above the median in adulthood.
- *Higher Income than Childhood* respondents have income above the median in adulthood but were raised in lower income households.
- *Lower Income than in Childhood* respondents grew up in higher income families but have lower income in adulthood.

#### Educational Attainment of Offspring

The classification of educational qualifications adopted by the HILDA Survey is based on the Australian Standard Classification of Education (ASCED) (44), which classifies formal educational qualifications by level and field of study. The highest educational attainment level is derived from information on the highest year of school completed and the level of highest non-school qualification. In this paper, up to four levels of attainment are distinguished: (1) Postgraduate degree (Masters or PhD) or Bachelor's degree, Graduate Diploma or Graduate Certificate (labeled as Degree+); (2) Diploma or Certificate Level 3 or 4 (Dip/Cert 3-4); (3) Completion of High School (Year 12); and (4) Non-completion of High School (Year 11 and below).

Additional variables including sex (female = 1) and age (squared) are included in the analysis. Descriptive statistics for all variables are included in Table 1.

**Table 1:** Descriptive statistics: proportion and means (and standard errors), weighted.

	**Mean/proportion**	**Standard errors**
**Offspring characteristics**		
Physical functioning – (ranked 1–100)	93.40	(0.31)
Mental health – (ranked 1–100)	72.20	(0.37)
Self-reported health status (QALY) – (ranked 1–100)	84.20	(0.30)
Female (*100)	0.50	(0.01)
Age	23.60	(0.05)
**Parents' characteristics**		
Physical functioning (both) – (ranked 1–100)	87.50	(0.33)
Mental health (both) – (ranked 1–100)	73.80	(0.31)
Self-reported health status (QALY) (both) – (ranked 1–100)	80.60	(0.31)
Father's age	46.20	(0.15)
Mother's age	43.60	(0.13)
**Early-life disadvantage**		
Father ever unemployed during childhood (*100)	0.06	(0.01)
Mother ever unemployed during childhood (*100)	0.10	(0.01)
Father less than high school (*100)	0.20	(0.01)
Mother less than high school (*100)	0.29	(0.01)
Neighborhood during childhood (based on the Socio-Economic Indexes for Areas-SEIFA)^a^ – (ranked 1–10)	5.92	(0.07)
**Offspring education**		
Year 11 and below (*100)	0.12	(0.01)
Year 12 (*100)	0.25	(0.01)
Dip/Cert 3-4 (*100)	0.27	(0.01)
Degree+ (*100)	0.36	(0.01)
Child health poor (*100)	0.02	(0.00)
Child health missing (*100)	0.01	(0.00)
Income comparison (*100)		
Stable low-income	0.25	(0.01)
Higher income than in childhood	0.17	(0.01)
Lower income than in childhood	0.19	(0.01)
Stable high-income	0.39	(0.01)
**Years of health measurements** ^**b**^		
Physical functioning (Offspring 18–35)	9.69	(0.10)
Mental health (Offspring 18–35)	9.72	(0.10)
Mental health (Offspring 18–35)	9.66	(0.10)
Physical functioning mother (Offspring <18)	5.52	(0.09)
Physical functioning father (Offspring <18)	5.07	(0.09)
Mental health mother (Offspring <18)	5.55	(0.09)
Mental health father (Offspring <18)	5.13	(0.09)
Self-reported health status (QALY) (Offspring <18)	5.50	(0.09)
Self-reported health status (QALY) (Offspring <18)	5.07	(0.09)
**Observations**	1,960	

*Individuals' cross-section sampling weights are used in this analysis*.

a *Socio-Economic Indexes for Areas rank from 1 to 10 with higher values associated with greater prosperity. For further information, see: Australian Bureau of Statistics (ABS) (44). Information Paper: An Introduction to Socio-Economic Indexes for Areas (SEIFA), Catalog No. 2309.0, ABS, Canberra*.

b *These variables show how many years of data on health measurements we have on average for respondents*.

## Results

The descriptive statistics for the sample are presented in Table 1. Approximately half of the sample is female. Offspring's physical health as measured by the SF-36 has mean of 93.60 compared to mean parental physical health of 87.50. Offspring mean mental health measured by the SF-36 is 72.20 compared to parental mental health of 73.80. Offspring mean QALY calculated using SAH is 84.20 and parents mean QALY is 80.60. It is interesting to note that offspring have better physical health than their parent's but slightly lower mental health. The mean age of parents is 46.20 for fathers and 43.60 for mothers and 23 for offspring. Approximately 6% of offspring had a father unemployed at some point during childhood and 10% of offspring had a mother who was unemployed at some point during childhood. 20% of fathers in the sample had less than high school education and 29% of mothers had less than high school education. This compares with 36% of offspring who had a university degree. 25% of offspring in the sample lived in households that fall below the median in adulthood and who resided in households with average income below the median as children. 17% of the sample had higher income as offspring than during childhood. 19% of the sample grew up in higher income families but have lower income in adulthood and 39% of the sample were raised in a household with income above the median and who on average have income above the median in adulthood.

To address RQ1, RQ2, and RQ3, we estimated a stepwise model. We began with a base model which included basic sociodemographic controls and parents' health as predictors of offspring health in young adulthood. We build upon the base model by adding variables related to early-life disadvantage (specification 2), childhood health (specification 3), educational attainment (specification 4) and income comparison in childhood and adulthood (specification 5). The results from these models are presented in Table 2.

**Table 2:** OLS regressions Offspring's health status as young adults on a set of covariates: Intergenerational health correlations.

	**Physical functioning**					**Mental health**					**Self-reported health status (QALY)**				
	**1**	**2**	**3**	**4**	**5**	**1**	**2**	**3**	**4**	**5**	**1**	**2**	**3**	**4**	**5**
**Offspring characteristics**															
Female	−0.94	−0.95	−0.94	−1.59^***^	−1.40^**^	−3.36^***^	−3.31^***^	−3.26^***^	−3.64^***^	−3.39^***^	−1.92^***^	−1.92^***^	−2.01^***^	−2.58^***^	−2.43^***^
	(0.58)	(0.58)	(0.58)	(0.60)	(0.59)	(0.63)	(0.63)	(0.64)	(0.65)	(0.64)	(0.51)	(0.50)	(0.50)	(0.49)	(0.49)
Age^2^	0.004	0.005	0.006^*^	0.006^*^	0.006^*^	0.0026	0.0021	0.0020	0.0016	0.0010	−0.012^***^	−0.011^***^	−0.011^***^	−0.011^***^	−0.012^***^
	(0.00)	(0.00)	(0.00)	(0.00)	(0.00)	(0.00)	(0.00)	(0.00)	(0.00)	(0.00)	(0.00)	(0.00)	(0.00)	(0.00)	(0.00)
**Parents' characteristics when child <18**															
Physical functioning (both)	0.19*** (0.03)	0.16*** (0.03)	0.15*** (0.03)	0.13*** (0.03)	0.13*** (0.03)										
Physical functioning missing	−1.87*** (0.70)	−2.33*** (0.72)	−2.35*** (0.73)	−1.70** (0.71)	−1.42** (0.71)										
Mental health (both)						0.21^***^	0.20^***^	0.19^***^	0.19^***^	0.18^***^					
						(0.02)	(0.03)	(0.03)	(0.03)	(0.03)					
Mental health missing						−1.15	−1.13	−1.04	−0.68	−0.17					
						(0.73)	(0.75)	(0.76)	(0.77)	(0.78)					
Self-reported health status both											0.20*** (0.03)	0.18*** (0.03)	0.15*** (0.02)	0.14*** (0.02)	0.14*** (0.02)
Self-reported health missing											−1.87*** (0.58)	−2.03*** (0.59)	−1.99*** (0.58)	−1.48** (0.58)	−1.37** (0.58)
(Age both)^2^	0.003^***^	0.002^***^	0.002^***^	0.002^***^	0.001^**^	0.0014^**^	0.0012^*^	0.0013^*^	0.00091	0.00063	0.0029^***^	0.0024^***^	0.0024^***^	0.0018^***^	0.0016^***^
	(0.00)	(0.00)	(0.00)	(0.00)	(0.00)	(0.00)	(0.00)	(0.00)	(0.00)	(0.00)	(0.00)	(0.00)	(0.00)	(0.00)	(0.00)
**Early-life disadvantage**															
Father ever unemployed during childhood		−0.19 (1.45)	−0.25 (1.47)	−0.33 (1.44)	0.06 (1.49)		−0.70 (1.51)	−0.67 (1.53)	−0.77 (1.52)	−0.27 (1.52)		0.55 (1.17)	0.43 (1.17)	0.47 (1.15)	0.54 (1.16)
Mother ever unemployed during childhood		0.66 (0.91)	0.62 (0.91)	1.11 (0.91)	1.33 (0.91)		−1.04 (1.21)	−1.25 (1.20)	−1.02 (1.21)	−0.63 (1.17)		−0.24 (0.93)	−0.33 (0.92)	0.02 (0.90)	0.04 (0.87)
Father less than high school		−2.58^***^	−2.603^***^	−1.95^**^	−1.71^**^		−0.74	−0.83	−0.43	0.012		−1.52^*^	−1.84^**^	−1.32^*^	−1.25^*^
		(0.93)	(0.91)	(0.92)	(0.93)		(0.97)	(0.98	(0.97)	(0.97)		(0.80)	(0.78)	(0.76)	(0.74)
Mother less than high school		−1.02 (0.77)	−1.01 (0.78)	−0.20 (0.75)	−0.13 (0.73)		0.91 (0.73)	0.64 (0.73)	1.09 (0.75)	1.19 (0.74)		−0.60 (0.60)	−0.55 (0.59)	0.081 (0.59)	0.11 (0.58)
Neighborhood during childhood (SEIFA)		0.27** (0.11)	0.25** (0.12)	0.15 (0.12)	0.07 (0.12)		0.17 (0.13)	0.16 (0.13)	0.10 (0.13)	−0.055 (0.13)		0.23** (0.11)	0.24** (0.11)	0.13 (0.11)	0.09 (0.11)
Child health poor			−5.83^**^	−4.79^*^	−4.65^*^			−7.78^***^	−7.19^***^	−6.85^***^			−9.04^**^	−8.44^***^	−8.39^***^
			(2.63)	(2.63)	(2.57)			(2.59)	(2.52)	(2.40)			(2.37)	(2.29)	(2.21)
**Offspring education**															
Year 11 and below				−7.78^***^	−6.87^***^				−4.16^***^	−2.77^**^				−5.18^***^	−4.44^***^
				(1.31)	(1.28)				(1.21)	(1.21)				(0.90)	(0.91)
Year 12				−2.11^***^	−1.74^**^				−1.67^*^	−1.08				−3.79^***^	−3.50^***^
				(0.73)	(0.72)				(0.87)	(0.86)				(0.69)	(0.67)
Dip/Cert 3-4				−2.94^***^	−2.49^***^				−1.09	−0.39				−3.88^***^	−3.48^***^
				(0.63)	(0.63)				(0.80)	(0.79)				(0.62)	(0.62)
**Social mobility**															
Stable low-income					−4.18*** (0.86)					−5.06*** (1.01)					−3.46*** (0.71)
Lower income than					−3.79^***^					−4.70^***^					−4.18^***^
childhood					(0.95)					(1.09)					(0.85)
Stable high-income					−1.11 (0.68)					0.12 (0.87)					−1.27* (0.61)
Constant	69.1^***^	71.4^***^	71.9^***^	78.0^***^	80.8^***^	53.9^***^	54.3^***^	54.9^***^	57.9^***^	61.5^***^	69.5^***^	70.7^***^	73.9^***^	79.1^***^	82.0^***^
	(3.56)	(3.59)	(3.59)	(3.45)	(3.41)	(3.14)	(3.27)	(3.31)	(3.39)	(3.45)	(3.13)	(3.24)	(2.83)	(2.87)	(2.95)
**Observations (*****n*** **= 1,960)**															
*R* ^2^	0.06	0.08	0.08	0.11	0.13	0.07	0.07	0.08	0.08	0.11	0.10	0.10	0.11	0.14	0.16

*Omitted categories are Degree+ for offspring education and higher income than childhood for social mobility*.

* *p < 0.10*,

** *p < 0.05*,

*** *p < 0.01*.

Our first research question estimates the intergenerational correlation in mental and physical health, and QALY. In our baseline specification (Specification 1), there is a significant intergenerational correlation between young adults and parents' health ranging from 0.19 for physical health, 0.21 for mental health and 0.20 for QALYs. In Specification 2, when we include variables related to early life disadvantage, the intergenerational correlation in physical health is 0.16, the intergenerational correlation in mental health is 0.20 and intergenerational correlation in QALYs is 0.18. In Specification 3, we included childhood health, and the intergenerational correlation in physical health is 0.15, mental health is 0.19, and QALY is 0.15. In Specification 4, we add in the educational attainment of the young adult offspring, and the intergenerational correlation in physical health is 0.13, mental health is kept at 0.19, and QALYs is now 0.14. In Specification 5, we included variables related to income comparisons in childhood and adulthood. The intergenerational correlation in physical health is 0.13, the intergenerational correlation in mental health is 0.18, and the intergenerational correlation in QALYs is unchanged.

Next, to address RQ2, we can say that after conditioning on early life disadvantage, the intergenerational correlation in health is reduced by 15.8% for the intergenerational correlation in physical health, 4.8% for the intergenerational correlation in mental health, and 10% for the correlation in QALYs^4^. When we include childhood health, it decreases by 6.2% for the intergenerational correlation in physical health, 5% for the intergenerational correlation in mental health and 16.7% for the intergenerational correlation in QALYs. Further, adding educational attainment of the young adult reduces the intergenerational correlation in health by 13.3% of the intergenerational correlation in physical, and by 6.7% for the intergenerational correlation in QALYs. Finally, in specification 5, differences in income in childhood and adulthood does not significantly change the intergenerational correlation in physical health or QALYs, but it further reduces by 5.2% the intergenerational correlation in mental health. Using all covariates together, the unconditional intergenerational persistence of health is reduced by 31.6% for physical functioning, by 14.3% for mental health and by 30% for QALYs. These results should be interpreted with caution as we cannot estimate causal relationships so endogeneity may bias our findings.

The significance and magnitude of the coefficients including to control for early life disadvantage, childhood health, educational attainment and income comparisons between childhood and adulthood follow our expectations regarding the factors that are significantly associated with health. Thus, children who grew up in a household where the father was less educated are more likely to report poorer physical health (coeff = −1.71) and poorer QALY (coeff = −1.25). In a similar manner, poor health in childhood, is significantly related to poorer health in all the dimensions measured: physical health (coeff = −4.65), mental health (coeff = −6.85) and QALY (coeff = −8.39). Likewise, results for young adults' educational attainment indicate that, compared to those with a university degree (reference category), lower levels of education are more likely to report poorer health. Coefficients are particularly large for physical health.

Lastly, results on income comparisons between childhood and adulthood reveal that compared to those with higher income than childhood, respondents with stable low-income are more likely to experience poor health for all the health measures used. The negative relationship is stronger for mental health (coeff = −5.06) and weaker for QALY (coeff = −3.46). Similarly, those with lower income than childhood are also more likely to experience poor health compared to those with higher income than childhood. These results suggest that having higher income as offspring compared to income during childhood offers some protection against reporting poor health in young adulthood.

We know that some factors might be considered pre-determined and exogenous, while others are clearly endogenous choices that are affected by parent and child health. Since our purpose is to study intergenerational health inequality, we consider these results as the first step for future research that may consider structural models to better understand the mechanisms that can identify causal channels.

## Discussion

We use data from the HILDA survey to explore the intergenerational correlation in mental and physical health measured using the SF-36 and QALYS calculated from a self-assessed health variable. Next, we assess the contribution of early life factors including deprivation, childhood health, social mobility, and educational attainment on the intergenerational correlation in health across the three different health measures.

In our base specification, we find an intergenerational correlation in health ranging from 0.19 for physical health to 0.20 for the QALY and 0.21 for mental health. Our findings are similar to those reported by Bencsik et al. (13) for the UK and are slightly lower than the correlations reported by Halliday et al. (12) for the USA. There are some similarities and differences between the USA, UK, and Australia. Australia and the USA are countries with large geographical land masses, have heterogeneous populations composed almost entirely of immigrants, commonalities in government systems and economic structure (16). The UK and Australia have highly ranked health care systems providing universal coverage to the population compared to the more fragmentary delivery system in the USA. Australia's life expectancy has continued to rise since 2010, where life expectancy has stagnated or fallen in the UK and USA, respectively (45). Australia has higher social mobility levels than both the USA and UK (46). Thus, comparing intergenerational correlations in Australia to findings from the USA and UK provides an interesting illustration of how differences in population health and mobility may impact correlations between families. Our results suggest a similar level of health mobility in Australia to that of the UK. The importance of universal provision of health care and a similar cultural heritage may explain this finding. We find a stronger correlation in mental health than Johnston et al. (11), which may reflect the data as Johnston uses a cohort survey from the South West of England or the different time period of the analysis. Our correlations in physical health are larger than those reported by Pascual and Cantarero (10). But, this may be explained by difference in the health measure or a different context as Pascual and Cantarero used data from Spain. This suggests context may be important. Further cross-country comparative analysis is needed to better understand how a country's macroeconomic and health conditions impact the intergenerational correlation in health to promote health mobility. Our findings support the importance of promoting good mental health as the impact of mental health can be felt across generations. If there are no measures to support good mental health in families, there may be a long tail of poor health felt for many years to come. Our results also provide further evidence on the importance of estimating separate correlations for physical and mental health as the intergenerational correlation is different for these two aspects of overall health.

We find that early life disadvantage is the only factor that has a larger impact decreasing the intergenerational correlation for all three health measures. Early life disadvantage and social mobility reduce to a large extent the intergenerational correlation in physical health. Early life disadvantage is the only set of variables that decreases the intergenerational correlation in mental health. Early life disadvantage, educational attainment, and early childhood health decrease by 20% of the intergenerational correlation in health as measured by QALYs. This builds upon the work of Halliday et al. (12) who investigate the contribution of ethnicity, education (of both parent and offspring) and income on the intergenerational correlation in health in the US. They find educational attainment to be the most important factor. These findings are different to what we found, in that in our results early life disadvantage was the most important factor. This divergence in results may be related to context and country-specific factors; but it warrants further investigation.

In terms of the policy implications of our research, our findings suggest that to promote health mobility it is important to focus on mental health. Policies that promote good mental health in families may have positive long-term consequences. Moreover, in Australia, combating early life disadvantage, in particular, through programmes such as creating safe green spaces in areas of disadvantage, job-seeking support, and tax credits for low-income families may be another mechanism to promote health mobility and reduce structural health inequalities.

## Data Availability Statement

This paper uses unit record data from the Household, Income and Labour Dynamics in Australia (HILDA) Survey. The HILDA Project was initiated and is funded by the Australian Government Department of Social Services (DSS) and is managed by the Melbourne Institute of Applied Economic and Social Research (Melbourne Institute). The findings and views reported in this paper, however, are those of the author and should not be attributed to either DSS or the Melbourne Institute. All data is available to download for users who register with Australian Data Archive. More information can be found at: https://dataverse.ada.edu.au/dataverse/hilda.

## Ethics Statement

The studies involving human participants were reviewed and approved by the Human Research Ethics Committee of the University of Melbourne. Written informed consent to participate in this study was provided by the participants' legal guardian/next of kin.

## Author Contributions

EV-T and HB planned the study, drafted, and edited the paper. EV-T conducted the data analysis and submitted the paper. Both authors contributed to the article and approved the submitted version.

## Conflict of Interest

The authors declare that the research was conducted in the absence of any commercial or financial relationships that could be construed as a potential conflict of interest.

## Publisher's Note

All claims expressed in this article are solely those of the authors and do not necessarily represent those of their affiliated organizations, or those of the publisher, the editors and the reviewers. Any product that may be evaluated in this article, or claim that may be made by its manufacturer, is not guaranteed or endorsed by the publisher.

## Footnotes

^1^In our sample, 68.9% are matched to both parents, 27.4% to the mother only and 3.7 % to the father only.

^2^The SF-36 physical functioning index was constructed using the individuals' responses to the following items: 1) vigorous activities, 2) moderate activities, 3) lifting or carrying groceries, 4) climbing several flights of stairs, 5) climbing one flight of stairs, 6) bending kneeling or stooping, 7) walking more than one kilometre, 8) walking half a kilometre, 9) walking 100 metres, 10) bathing or dressing yourself.

^3^Estimations at the baseline specification include as controls the quadratic age terms of the mother, father and child, and missing indicators for mother and father, as well as a dummy for female. Age for both generations is defined as the average age of the individual at the time of health observations.

^4^It is important to bear in mind that we do not calculate confidence intervals for the change in percentages from adding additional variables to our estimation models. Thus, though we observe a decrease in the magnitude of the coefficient, we cannot be sure that this is a statistically significant difference.
